# Increased intelligence is a myth (so far)

**DOI:** 10.3389/fnsys.2014.00034

**Published:** 2014-03-12

**Authors:** Richard J. Haier

**Affiliations:** Emeritus, Pediatrics, School of Medicine, University of CaliforniaIrvine, CA, USA

**Keywords:** intelligence, *g*-factor, brain imaging, cognitive training, ratio scales, IQ testing

On one hand, intelligence testing is one of the great successes of psychology (Hunt, 2011). Intelligence test scores predict many real world phenomena and have many well-validated practical uses (Gottfredson, 1997; Deary et al., 2010). Intelligence test scores also correlate to structural and functional brain parameters assessed with neuroimaging (Haier et al., 1988; Jung and Haier, 2007; Deary et al., 2010; Penke et al., 2012; Colom et al., 2013a) and to genes (Posthuma et al., 2002; Hulshoff Pol et al., 2006; Chiang et al., 2009, 2012; Stein et al., 2012). On the other hand, intelligence test scores are often misunderstood and can be misused. This paper focuses on a basic misunderstanding that permeates many of the recent reports of increased intelligence following short-term cognitive training. Several of these reports have been published in prominent journals and received wide public attention (Jaeggi et al., 2008, 2011; Mackey et al., 2011).

The basic misunderstanding is assuming that intelligence test scores are units of measurement like inches or liters or grams. They are not. Inches, liters and grams are ratio scales where zero means zero and 100 units are twice 50 units. Intelligence test scores estimate a construct using interval scales and have meaning only relative to other people of the same age and sex. People with high scores generally do better on a broad range of mental ability tests, but someone with an IQ score of 130 is not 30% smarter then someone with an IQ score of 100. A score of 130 puts the person in the highest 2% of the population whereas a score of 100 is at the 50th percentile. A change from an IQ score from 100 to 103 is not the same as a change from 133 to 136. This makes simple interpretation of intelligence test score changes impossible.

Most recent studies that have claimed increases in intelligence after a cognitive training intervention rely on comparing an intelligence test score before the intervention to a second score after the intervention. If there is an average change score increase for the training group that is statistically significant (using a dependent t-test or similar statistical test), this is treated as evidence that intelligence has increased. This reasoning is correct if one is measuring ratio scales like inches, liters or grams before and after some intervention (assuming suitable and reliable instruments like rulers to avoid erroneous Cold Fusion-like conclusions that apparently were based on faulty heat measurement); it is not correct for intelligence test scores on interval scales that only estimate a relative rank order rather than measure the construct of intelligence. Even though the estimate has considerable predictive value and correlates to brain and genetic measures, it is not a measurement in the same way we measure distance, liquid, or weight even if individual change scores are used in a pre-post design.

SAT scores, for example, are highly correlated to intelligence test scores (Frey and Detterman, 2004). Imagine a student takes the SATs when quite ill. The scores likely are a bad estimate of the student's ability. If the student retakes the test sometime later when well, does an increase in score mean the student's intelligence has increased, or that the newer score is now just a better estimate? The same is true for score changes following SAT preparatory courses. Many colleges and universities allow applicants to submit multiple SAT scores and the highest score typically carries the most weight; there are many spurious reasons for low scores but far fewer for high scores. Change scores from lowest to highest carry little if any weight. By contrast, change in a person's weight after some intervention is unambiguous.

In studies of the effect of cognitive training on intelligence, it is also important to understand that all intelligence test scores include a certain amount of imprecision or error. This is called the standard error of measurement and can be quantified as an estimate of a “true” score based on observed scores. The standard error of measuring inches or liters is usually zero assuming you have perfectly reliable, standard measurement devices. Intelligence tests generally show high test-retest reliability but they also have a standard error, and the standard error is often larger for higher scores than for lower scores. Any intelligence test score change after an intervention needs to be considered relative to the standard error of the test. Studies that use a single test to estimate intelligence before and after an intervention are using less reliable and more variable scores (bigger standard errors) than studies that combine scores from a battery of tests.

Change scores are never easy to interpret and require sophisticated statistical methods and research designs with appropriate control groups. If you try a training intervention in individuals all of whom have pre-intervention scores below the population mean, for example, re-testing with or without any intervention, may result in higher scores due to the statistical phenomenon of regression to the mean, or due to simple test practice, especially if equivalent alternative forms of the test are not used. Quasi-experimental designs like post-test only with large samples and random assignment do not have all the same interpretation difficulties as pre-post designs. They have promise but most reviewers are more inclined to value pre-post changes. Latent variable techniques also avoid many of the difficulties of pre-post interval scale changes and they have promise in large samples (Ferrer and McArdle, 2010).

When change scores are used, it is important to identify individual differences even within a group where the average change score statistically increases after an intervention. Imagine a group of 100 students received cognitive training and 100 others received some control intervention. The mean change score in the training group may statistically show a greater increase than the controls. How many of the 100 individuals who received the training actually show an increase? Do they differ in any way from the individuals in the same group who do not show an increase? Does item analysis show whether increased scores are due more to easy test items or hard ones? What about any individuals in the control group that show change score increases as large as shown in the training group? If all 200 participants ultimately get the same training, will the rank order of individuals based on the post-training score be any different than the rank order based on the pre-training scores? If not, what has been accomplished? Most studies do not report such analyses, although newer training studies are addressing issues of multiple measure assessment of intelligence and individual differences (Colom et al., 2013b; Jaeggi et al., 2013). Burgaleta et al provide a good example of showing IQ changes subject-by-subject (Burgaleta et al., 2014).

Nonetheless, the main point is that to make the most compelling argument that intelligence increases after an intervention, a ratio scale of intelligence is required. None yet exists and meaningful progress may require a new way of defining intelligence based on measureable brain or information processing variables. For example, gray and white matter density in specific brain regions assessed by imaging and expressed as a profile of standard scores based on a normative group might substitute for intelligence test scores (Haier, 2009). Work by Engle and colleagues suggests that working memory capacity and perceptual speed are possible ways to assess fluid intelligence (Broadway and Engle, 2010; Redick et al., 2012) based on a large body of research that shows faster mental processing speed and increased memory capacity are related to higher intelligence.

Jensen has written extensively about an evolution from psychometrics to mental “chronometrics”—the use of response time in milliseconds to measure information processing in a standard way (Jensen, 2006). He argued that the construct of intelligence could be replaced in favor of ratio scale measures of speed of information processing assessed during standardized cognitive tasks like the Hick paradigm. Such measures, for example, would help advance research about the underlying neurophysiology of mental speed and might lead to a more advanced definition of intelligence. Jensen concluded his book on chronometry with this call to action: “… chronometry provides the behavioral and brain sciences with a universal absolute scale for obtaining highly sensitive and frequently repeatable measurements of an individual's performance on specially devised cognitive tasks. Its time has come. Let's get to work!” (p. 246).

This is a formidable challenge and a major priority for intelligence researchers. Collaboration among psychometricians and cognitive psychologists will be key. There are now a number of studies that fail to replicate the claims of increased intelligence after short-term memory training and various reasons are proposed (Colom et al., 2013b; Harrison et al., 2013). Given our narrow focus here, we note one failure to replicate also assessed working memory capacity and perceptual speed; no transfer effects were found (Redick et al., 2013) and there is reason to suggest that other positive transfer studies may be erroneous (Tidwell et al., 2013). For now, cognitive training results are more inconsistent than not, especially for putative intelligence increases. Nonetheless, it is encouraging that cognitive researchers are working on these issues despite a pervasive indifference or negativity to intelligence research in Psychology in general and for many funding agencies.

In the broader context, intelligence includes more than one component. However, the construct of interest usually is defined by psychometric methods as a general factor common to all mental abilities called the *g*-factor (Jensen, 1998). Fluid intelligence, the focus of several cognitive training studies, is one of several broad intelligence factors and it is highly correlated to *g*. The *g*-factor is estimated by intelligence tests but it is not synonymous with IQ or any other test score; some tests are more *g*-loaded than others. As noted, a score on an intelligence test has little meaning without comparing it to the scores of other people. That is why all intelligence tests require normative groups for comparison and why norm groups need to be updated periodically, as demonstrated by the Flynn Effect of gradual generational increases in intelligence test scores; although whether *g* shows the Flynn effect is still unsettled (te Nijenhuis and van der Flier, 2013). Psychometric estimations of *g* and other intelligence factors have generated strong empirical findings about the nature of intelligence and individual differences, mostly based on correlation studies. These interval assessments, however, are not sufficient to take research to the next step of experimental interventions to increase intelligence.

Speaking about science, Carl Sagan observed that extraordinary claims require extraordinary evidence. So far, we do not have it for claims about increasing intelligence after cognitive training or, for that matter, any other manipulation or treatment, including early childhood education. Small statistically significant changes in test scores may be important observations about attention or memory or some other elemental cognitive variable or a specific mental ability assessed with a ratio scale like milliseconds, but they are not sufficient proof that general intelligence has changed. As in all branches of science, progress depends on ever more sophisticated measurement that drives more precise definitions—think about the evolution of definition for a “gene” or an “atom”. Even with sophisticated interval-based assessment techniques (Ferrer and McArdle, 2010), until we have better measures, especially ratio scales, we need to acknowledge the basic measurement problem and exercise abundant restraint when reporting putative intelligence increases or decreases.

In the future, there may be strong empirical rationales for spending large sums of money on cognitive training or other interventions aimed at improving specific mental abilities or school achievement (in addition to the compelling moral arguments to do so), but increasing general intelligence is quite difficult to demonstrate with current tests. Increasing intelligence, however, is a worthy goal that might be achieved by interventions based on sophisticated neuroscience advances in DNA analysis, neuroimaging, psychopharmacology, and even direct brain stimulation (Haier, 2009, 2013; Lozano and Lipsman, 2013; Santarnecchi et al., 2013; Legon et al., 2014). Developing equally sophisticated ratio measurement of intelligence must go hand-in-hand with developing promising interventions.
